# *Bacillus subtilis* variant *natto* Bacteremia of Gastrointestinal Origin, Japan

**DOI:** 10.3201/eid2808.211567

**Published:** 2022-08

**Authors:** Ippei Tanaka, Satoshi Kutsuna, Misako Ohkusu, Tomoyuki Kato, Mari Miyashita, Ataru Moriya, Kiyofumi Ohkusu

**Affiliations:** Japanese Red Cross Musashino Hospital, Tokyo, Japan (I. Tanaka, T. Kato, M. Miyashita);; Osaka University, Osaka, Japan (S. Kutsuna); Chiba University, Chiba, Japan (M. Ohkusu);; National Center for Global Health and Medicine, Tokyo (A. Moriya);; Tokyo Medical University, Tokyo (K. Ohkusu)

**Keywords:** Bacillus subtilis var. natto, bacteremia, gastrointestinal origin, bacteria, Japan

## Abstract

We report a case of bacteremia caused by *Bacillus subtilis* variant *natto* after a gastrointestinal perforation in a patient in Japan. Genotypic and phenotypic studies of biotin identified *B. subtilis* var. *natto*. This case and 3 others in Japan may have been caused by consuming natto (fermented soybeans).

*Bacillus subtilis* is a gram-positive, rod-shaped, spore-forming bacterium temporarily present in the human gastrointestinal tract (*1*). The presence of *B. subtilis* in clinical specimens indicates contamination, but rare cases of bacteremia have been reported in Japan (*2*). Previous reports have attributed bacteremia in Japan to gastrointestinal origin but of unknown cause. We identified a case of *B. subtilis* variant *natto* bacteremia in a patient in Japan.

In May 2021, a 56-year-old woman was referred to the Japanese Red Cross Musashino Hospital (Musashino-shi, Tokyo, Japan) for a 2-day history of abdominal pain after having taken barium for gastric radiographic examination. The patient had a history of hypertension and ate natto (fermented soybeans) almost every day. At admission, the patient exhibited spontaneous abdominal pain, muscular defense, and rebound tenderness. Laboratory findings showed a decreased leukocyte count (1,800 cells/µL, reference range 3,300–8,600 cells/µL) and mildly increased C-reactive protein concentration (0.75 mg/dL, reference range 0–0.14 mg/dL). Contrast-enhanced computed tomography revealed contrast accumulation in the colon and free air around the sigmoid rectum. Lower gastrointestinal perforation and generalized peritonitis were suspected, and 2 sets of blood cultures were obtained. Emergency proctosigmoidectomy (Hartmann surgery) was performed on the same day, and perforation of the sigmoid colon was confirmed.

Intravenous antimicrobial treatment was initiated. Initial treatment was piperacillin/tazobactam (18 g/d). On day 5, because both blood culture sets were positive for gram-positive rod bacteria, teicoplanin (800 mg/d) was added. On day 11, only *B. subtilis* was isolated from the culture by matrix-assisted laser desorption/ionization-time of flight mass spectrometry, and the antimicrobial drugs were changed to ampicillin/sulbactam (12 g/d) as indicated by antimicrobial susceptibility testing by broth microdilution (Appendix Table). *B. subtilis* was also detected along with multiple other bacteria by culture of ascites fluid collected intraoperatively. After 39 days of antimicrobial therapy, the patient was discharged.

We investigated whether the blood culture isolate was *B. subtilis* var. *natto*. DNA analysis showed that in the *bioF* region, the isolate was 100% homologous to the *B. subtilis* var. *natto* standard strain. Compared with the *B. subtilis* subspecies *subtilis* standard strain, the isolate had ≈50 fewer bases and the *bioW* region of the isolate had a single-nucleotide mutation that resulted in a termination codon for amino acid synthesis (Appendix Figures 1–4). The isolate and *B. subtilis* var. *natto* standard strain grew abundantly on a biotin-supplemented medium but did not thrive on a nonsupplemented medium (Figure).

**Figure:**
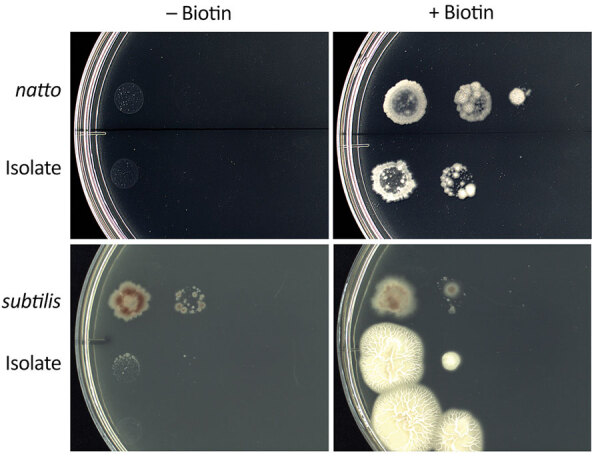
*Bacillus subtilis* cultures on E9 minimal medium agar plates with and without biotin. From left to right in each column, 0.5 McFarland standard was diluted ×1, ×10, and ×10^2^, and 10 μL was incubated at 35°C for 72 hours under aerobic conditions. The isolate showed a biotin requirement. Isolate, *Bacillus subtilis* variant *natto* from patient in Japan with bacteremia of gastrointestinal origin; *natto*, *B. subtilis* var. *natto* standard strain; subtilis, *B. subtilis* subspecies *subtilis* standard strain.

Our biotin gene and biotin requirement testing confirmed that the isolate in this case was *B. subtilis* var. *natto*. Previous genotypic and phenotypic studies on biotin were helpful in identifying this variant. Kubo et al. reported that natto-fermented *B. subtilis* requires biotin and that nonfermented *B. subtilis* does not (*3*). *bioF* and *bioW* are biotin biosynthetic operons in *B. subtilis* (*4*). Compared with the *B. subtilis* subsp. *subtilis* standard strain, the 2 biotin genes of the isolate in this study and the *B. subtilis* var. *natto* standard strain were partially defective. According to the biotin requirement test, the isolate required biotin.

We conclude that this case of bacteremia caused by *B. subtilis* var. *natto* resulted from a gastrointestinal perforation. In Japan, the most common causative organism of community-acquired bloodstream infections is gram-negative *Escherichia coli* (25.4%); gram-positive bacilli rarely induce bacteremia (2.7%) (*5*). *B. subtilis* bacteremia typically originates from the gastrointestinal tract (*2*); Tamura et al. have reported 3 cases of *B. subtilis* bacteremia arising from the gastrointestinal tract (*6*). In patients with gastrointestinal bacteremia, the causative organism differs according to the food consumed (*7*). Oggioni et al. reported a case of *B. subtilis* bacteremia caused by probiotics (*8*). However, the patient that we report was not taking any probiotics but frequently ate natto. Most of the previously reported cases of *B. subtilis* bacteremia in Japan (*2*,*6*) were possibly related to natto consumption, although dietary history was not mentioned in their reports.

This case of bacteremia caused by *B. subtilis* var. *natto* resulted from gastrointestinal tract perforation. Genotypic and phenotypic studies on biotin effectively identified *B. subtilis* var. *natto*. In Japan, natto consumption is common, and *B. subtilis* bacteremia of gastrointestinal origin is most likely associated with *B. subtilis* var. *natto*.

Appendix Supplemental results from study of patient with *Bacillus subtilis* variant *natto* bacteremia of gastrointestinal origin, Japan.
