# Supplementary material for: Bacillus subtilis variant natto Bacteremia of Gastrointestinal Origin, Japan
Source: Emerg Infect Dis. 2022 Aug;28(8):1718–9. doi: 10.3201/eid2808.211567 (PMC9328918; doi:10.3201/eid2808.211567)
Supplement: Appendix — Supplemental results from study of patient with Bacillus subtilis variant natto bacteremia of gastrointestinal origin, Japan. [file 21-1567-Techapp-s1.pdf]

Article DOI: https://doi.org/10.3201/eid2808.211567

# *Bacillus subtilis* variant *natto* Bacteremia of Gastrointestinal Origin, Japan

## Appendix

**Appendix Table.** Minimum inhibitory concentrations of each antimicrobial agent and their interpretation against the *Bacillus subtilis* var. *natto* strains isolated from the patient*

| Antimicrobial agent | MIC (µg/mL) | Interpretation† |
|---|---|---|
| Penicillin G | ≦0.12 | S |
| Ampicillin | ≦0.06 | S |
| Imipenem | ≦1 | S |
| Gentamicin | ≦1 | S |
| Erythromycin | ≦0.25 | S |
| Clindamycin | ≦0.25 | S |
| Levofloxacin | ≦0.5 | S |

*MIC, minimum inhibitory concentration; S, susceptible; R, resistant.
†Interpretation (susceptible, intermediate, or resistant) was based on the Clinical and Laboratory Standards Institute criteria (M45).

```
The isolate    1 GTGTTTATCCTGACATTGTGATCGGCACCTTAAGCAAAGCTGTTGGTACGGAAGGAGGTT  60
                 ||||||||||||||||||||||||||||||||||||||||||||||||||||||||||||
natto          1 GTGTTTATCCTGACATTGTGATCGGCACCTTAAGCAAAGCTGTTGGTACGGAAGGAGGTT  60

The isolate   61 TTGCGGCTGGATCAGCGGTCTTCATCGACTTTTTGCTGAACCATGCCAGAACATTTATCT 120
                 ||||||||||||||||||||||||||||||||||||||||||||||||||||||||||||
natto         61 TTGCGGCTGGATCAGCGGTCTTCATCGACTTTTTGCTGAACCATGCCAGAACATTTATCT 120

The isolate  121 TTCAAACCGCTATTCCGCCAGCCAGCTGTGCGGCTGCTCACGAGGCTTTCAACATCAGAA 180
                 ||||||||||||||||||||||||||||||||||||||||||||||||||||||||||||
natto        121 TTCAAACCGCTATTCCGCCAGCCAGCTGTGCGGCTGCTCACGAGGCTTTCAACATCAGAA 180

The isolate  181 CCAGTTTGAAGAATATGGGTTATGTGGTGAAAGGAGATCACACACCGATTATTCCTGTAG 240
                 ||||||||||||||||||||||||||||||||||||||||||||||||||||||||||||
natto        181 CCAGTTTGAAGAATATGGGTTATGTGGTGAAAGGAGATCACACACCGATTATTCCTGTAG 240

The isolate  241 TCATTGGCGATGCCCATAAAACGGTCATATTTGCTGAAAAACTGCAGGGCAAGGGAATTT 300
                 ||||||||||||||||||||||||||||||||||||||||||||||||||||||||||||
natto        241 TCATTGGCGATGCCCATAAAACGGTCATATTTGCTGAAAAACTGCAGGGCAAGGGAATTT 300

The isolate  301 ATGCTCCTGCCATTCGGCCGCCGACCGTTGCGCCGGGTGAAAG 343
                 |||||||||||||||||||||||||||||||||||||||||||
natto        301 ATGCTCCTGCCATTCGGCCGCCGACCGTTGCGCCGGGTGAAAG 343
```

**Appendix Figure 1.** The isolate showed 100% homology to *Bacillus subtilis* var. *natto* standard strain in the *bioF* sequence. natto, *Bacillus subtilis* var. *natto* standard strain.

```
The isolate    1 GTGTTTATCCTGACATTGTGATCGGCACCTTAAGCAAAGCTGTTGGTACGGAAGGAGGTT   60
                 |||||| |||||||||||||||||||||||||||||| ||||||||  ||||||||||||
subtilis       1 GTGTTTGTCCTGACATTGTGATCGGCACCTTAAGCAAGGCTGTTGGCGCGGAAGGAGGTT   60

The isolate   61 TTGCGGCTGGATCAGCGGTCTTCATCGACTTTTTGCTGAACCATGCCAGAACATTTATCT  120
                 ||||||| ||||||||||||||||||||||||||||||||||||||||||||||||||||
subtilis      61 TTGCGGCAGGATCAGCGGTCTTCATCGACTTTTTGCTGAACCATGCCAGAACATTTATCT  120

The isolate  121 TTCAAACCGCTATTCCGCCAGCCAGCTGTGCGGCTGCTCACGAGGCTTTCAACATCA  177
                 |||||||||||||||||||||||||||||||||||||||||||||||||||||||||
subtilis     121 TTCAAACCGCTATTCCGCCAGCCAGCTGTGCGGCTGCTCACGAGGCTTTCAACATCA  177


The isolate  174 ATCAGAACCAGTTTGAAGAATATGGGTTATGTGGTGAAAGGAGATCACACACCGATTATT  233
                 |||||||||||| ||||||||||||||||||| |||||||||||||||||||||||||||
subtilis     228 ATCAGAACCAGTCTGAAGAATATGGGTTATGTCGTGAAAGGAGATCACACACCGATTATT  287

The isolate  234 CCTGTAGTCATTGGCGATGCCCATAAAACGGTCATATTTGCTGAAAAACTGCAGGGCAAG  293
                 || || ||||||||||||||||||||||||||| ||||||||||||||||||||||||||
subtilis     288 CCCGTGGTCATTGGCGATGCCCATAAAACGGTCCTATTTGCTGAAAAACTGCAGGGCAAG  347

The isolate  294 GGAATTTATGCTCCTGCCATTCGGCCGCCGACCGTTGCGCCGGGTGAAAG  343
                 ||||||| ||||||||||||||||||||||||||||||||| ||||||||
subtilis     348 GGAATTTTTGCTCCTGCCATTCGGCCGCCGACCGTTGCGCCAGGTGAAAG  397
```

**Appendix Figure 2.** The isolate showed a 50-nt deletion in the *bioF* sequence compared to *Bacillus subtilis* subsp. *subtilis* standard strain. subtilis, *Bacillus subtilis* subsp. *subtilis* standard strain.

```
The isolate    1 TGCGCTCGCTTCAAAGGTAAGCCGGCACCCGGCAGCCATTGCAGAATTATGCTGGTCGGA   60
                 ||||||||||||||||||||||||||||||||||||||||||||||||||||||||||||
natto          1 TGCGCTCGCTTCAAAGGTAAGCCGGCACCCGGCAGCCATTGCAGAATTATGCTGGTCGGA   60

The isolate   61 CGATCCGGATTACATAACAGGCTATGTTGCGGGTAAGAAAATGGGCTATCAGCGTATTAC  120
                 ||||||||||||||||||||||||||||||||||||||||||||||||||||||||||||
natto         61 CGATCCGGATTACATAACAGGCTATGTTGCGGGTAAGAAAATGGGCTATCAGCGTATTAC  120

The isolate  121 AGCAATGAAAGAATACGGGACTGAAGAGGGCTGACGAGTCTTTTTTATTGATGGATCCAA  180
                 ||||||||||||||||||||||||||||||||||||||||||||||||||||||||||||
natto        121 AGCAATGAAAGAATACGGGACTGAAGAGGGCTGACGAGTCTTTTTTATTGATGGATCCAA  180

The isolate  181 TGATGTAAACACGTACATACATGATCTGGAGAAGCAGCCTATTTTAATAGAGTGGGAGG   239
                 |||||||||||||||||||||||||||||||||||||||||||||||||||||||||||
natto        181 TGATGTAAACACGTACATACATGATCTGGAGAAGCAGCCTATTTTAATAGAGTGGGAGG   239
```

**Appendix Figure 3.** The isolate showed 100% homology to *Bacillus subtilis* var. *natto* standard strain in the *bioW* sequence. natto, *Bacillus subtilis* var. *natto* standard strain.

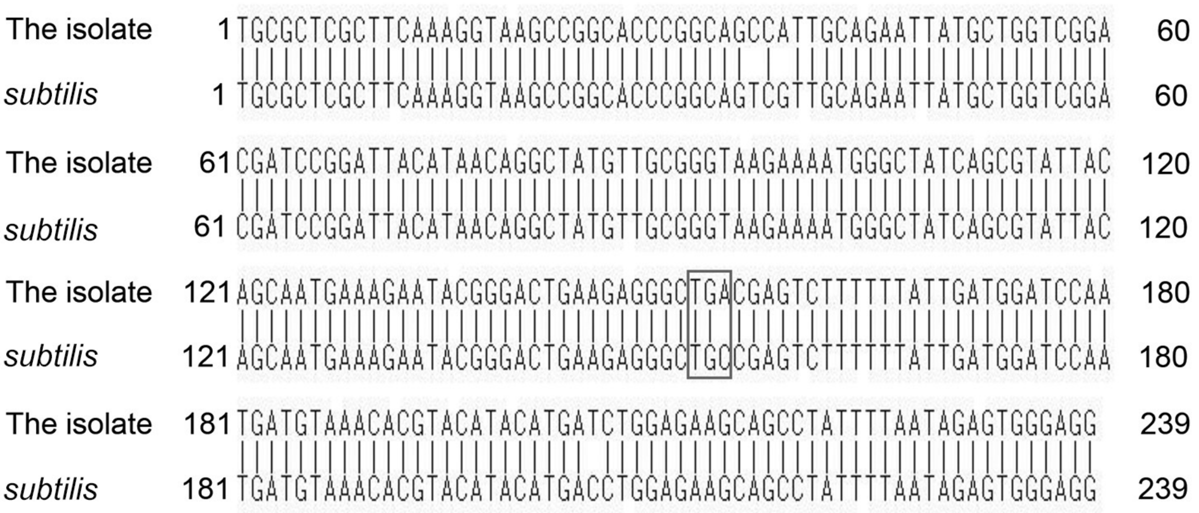


**Appendix Figure 4.** The isolate showed a single nucleotide mutation that resulted in a termination codon and stopped amino acid synthesis compared to *B. subtilis* subsp. *subtilis* standard strain in the *bioW* sequence. subtilis, *Bacillus subtilis* subsp. *subtilis* standard strain.
